# Generation of a BAC transgenic mouse strain that expresses CreERT and a fluorescent protein under the transcriptional control of the *Fzd5* locus

**DOI:** 10.1186/s41232-022-00194-x

**Published:** 2022-03-01

**Authors:** Satoru Miyagi, Yuko Kato, Ayako Watanabe, Kenichi Miyamoto, Rintaro Yoshikawa, Keita Hagiya, Daisuke Hirano, Yumi Matsuzaki

**Affiliations:** 1 Department of Life Science, Faculty of Medicine, Shimane University, 89-1 Enya, Izumo, Shimane 693-8501 Japan; 2 PuREC Co., Ltd., Izumo, Shimane Japan; 3 FUJIFILM Corporation, Minato-ku, Tokyo, Japan

**Keywords:** Mesenchymal stem/stromal cells (MSC), Fzd5, PαS, Leptin receptor-expressing MSC (LepR^+^MSC)

## Abstract

**Background:**

The expression of *FZD5* distinguishes immature human mesenchymal stem/stromal cells (MSC) in cultures, and the function of FZD5 is crucial for maintaining the proliferation and multilineage differentiation capacity of human MSC. We herein investigated whether *Fzd5* expression also marks undifferentiated MSC in animals.

**Methods:**

We generated a transgenic mouse strain (*Fzd5-CreERT-tFP635*) that expresses *CreERT* and the fluorescent protein, *TurboFP635* (*tFP635*), under the transcriptional control of the *Fzd5* gene using the BAC transgenic technique, and identified cells expressing tFP635 by flow cytometry. We also conducted lineage tracing with this strain.

**Results:**

In the bone marrow of transgenic mice, tFP635 was preferentially expressed in MSC, Leptin receptor-expressing MSC (LepR^+^MSCs), and some Pdgfrα^+^ Sca1^+^ MSC (PαS). Inducible lineage tracing using the *Fzd5-CreERT-tFP635*; *CAG-CAT-EGFP* strain at the adult stage showed that *Fzd5*-expressing cells and their descendants labeled with GFP were progressively dominant in LepR^+^MSC and PαS, and GFP^+^ cells persisted for 1 year after the activation of CreERT. Adipocyte progenitor cells (APCs), osteoblast progenitor cells (OPCs), and Cd51^+^ stromal cells were also labeled with GFP.

**Conclusions:**

Our transgenic mouse marks two different types of MSC, LepR^+^MSC and PαS.

**Supplementary Information:**

The online version contains supplementary material available at 10.1186/s41232-022-00194-x.

## Background

Mesenchymal stem/stromal cells (MSCs) self-renew and differentiate into mesenchymal lineages and reside in the bone marrow (BM) and other tissues. MSC have been isolated by plastic adherence and subsequent cultivation since the 1970s [1, 2]. In the last two decades, researchers have developed a strategy to prospectively isolate MSC from mouse BM and other tissues using monoclonal antibodies and/or reporter mice and identified various MSC fractions in vivo [3]. BM reticular cells expressing high levels of CXCL12 have been identified as CXCL12-abundant reticular cells (CAR cells), which preserve their self-renewal capacity and multilineage differentiation capacity in vitro and exhibit a lifelong self-renewal capacity in vivo. Histochemical analyses and cell ablation experiments revealed that CAR cells are an important source of niche factors that are required for the maintenance of hematopoietic stem cells (HSCs) [4, 5]. BM stromal cells, which express the leptin receptor (LepR^+^MSC), contribute to adipogenesis and osteogenesis in BM [6, 7]. LepR^+^MSC is also a component of the perisinusoidal niche and express the niche factors, *Kitl* and *Cxcl12*. Functionally, the conditional deletion of *Kitl* or *Cxcl12* in LepR^+^MSC leads to the depletion or mobilization of HSC, respectively, in BM. Therefore, LepR^+^MSC is critical for the maintenance of HSC and retention of HSC in the BM. A recent comparative analysis revealed that CAR cells and LepR^+^MSCs were substantially overlapping populations [8–10]. Cd51^+^Pdgfrα^+^ BM stromal cells form mesenspheres with a capacity for mesenchymal lineage differentiation. NG2^+^ *Nestin-GFP*^bright^ periarteriolar cells also maintain MSC properties, and Cd51^+^Pdgfrα^+^ stromal cells are a subpopulation of *Nestin-GFP*^+^ cells [11]. On the other hand, cultured MSCs exert immunomodulatory functions through various pathways and have been applied to clinical treatments [12, 13]. Accordingly, beyond the homeostasis of the mesenchymal cell population, BM-MSC are involved in the regulation of hematopoiesis and immune responses through the secretion of various cytokines and chemokines.

To date, various MSC fractions have been identified in mouse BM, and the function(s) of each BM-MSC are being revealed. However, many questions remain unanswered in MSC biology, such as whether known MSCs originate from a common “*bona fide* undifferentiated MSC,” if a hierarchical relationship exists between known MSCs, and how the proliferation, differentiation, and function(s) of each MSC are controlled. We previously demonstrated that mouse BM stromal cells expressing Pdgfrα and Sca1, named PαS, are highly enriched MSC with a high proliferative capacity and differentiation potential toward a mesenchymal lineage in vitro and in the transplantation setting [14]. We recently performed extensive gene expression profiling on human MSC clones and showed that *Frizzled5* (*FZD5*), a receptor for the WNT signaling molecule, was exclusively expressed in highly functional and immature human MSC clones. Gain- and loss-of-function analyses revealed that FZD5 negatively regulated cellular senescence in human MSC, thereby maintaining proliferation and multilineage potency [15].

To investigate whether the expression of *Fzd5* marks immature MSC in mouse, we herein generated a reporter mouse strain (*Fzd5-CreERT-tFP635*) that expresses CreERT and fluorescent protein, TurboFP635 (tFP635), under the control of the transcriptional regulatory sequence of the *Fzd5* gene using BAC transgenic technology. In the BM of transgenic mice, tFP635 is preferentially expressed in LepR^+^MSC and a part of PαS. A lineage tracing experiment with *Fzd5-CreERT-tFP635*; *CAG-CAT-EGFP* showed that LepR^+^MSC and PαS were labeled with GFP, the frequency of GFP^+^ cells in these fractions increased over time after the activation of CreERT, and GFP^+^ cells were maintained for up to 1 year, showing that our transgenic mouse marks two types of MSC, LepR^+^MSC, and PαS.

## Methods

### Generation of BAC transgenic mice

*Fzd5-CreERT-tFP635* were generated using the BAC transgenic technique. The coding sequence of the *Fzd5* gene in the BAC clone, RP23-142C12, was replaced with a 4.3 Kb DNA fragment containing *CreERT* and three copies of *tFP635* cDNA in *Escherichia coli* using the Red/ET Recombination System (Gene Bridges). Recombined BAC DNA was then injected into pronuclei and transplanted into pseudopregnant females. Ten independent founder lines were obtained after screening 60 F0 mice with Southern blotting using the Cre cDNA probe, and the lines A, F, G, H, and I were described herein.

### Mice

B6.129(Cg)-*Lepr*^*tm2(cre)*^*Rck*/J (*LepR-Cre*, JAX lab #008320) and B6.Cg-*Gt(ROSA)26Sor*^*tm14(CAG-tdTomato)Hze*^/J (*ROSA-CAG-LSL-tdTomato*, JAX lab #007914) were purchased from the Jackson Laboratory and backcrossed at least six times onto a C57BL/6J background. *CAG-CAT-EGFP* mice [16] were kindly provided by Dr. Jun-ichi Miyazaki (Osaka University, Osaka, Japan). All mice were maintained in the C57BL6J background.

In lineage tracing experiments, *Fzd5-CreERT-tFP635* or *LepR-Cre* were crossed with *CAG-CAT-EGFP* or *ROSA*-*CAG-LSL-tdTomato*, respectively, and compound mice were obtained. To induce CreERT activity in *Fzd5-CreERT-tFP635*; *CAG-CAT-EGFP*, 8- to 12-week-old mice were intraperitoneally injected with 100 μl of tamoxifen (TM) dissolved in corn oil at a concentration of 10 mg/ml for 3 or 5 consecutive days.

### Preparation of femur sections

The femur was cryosectioned using the Kawamoto film method [17].

### Preparation of BM cells for FCM and cell sorting

PαS is enriched in collagenase-released cells (CR)—the cells released from bone fragments with collagenase digestion [14]. In contrast, LepR^+^MSC comprise some reticular cells and reside in BM. Therefore, a femur and tibia pair was processed to prepare a single-cell suspension containing CR for the detection of PαS, and the other pair was used to prepare BM and detect LepR^+^MSC. To prepare the single-cell suspension for PαS staining, the femur and tibia were minced with scissors and incubated at 37°C for 30 min in collagenase solution [DMEM (Wako Chemicals) containing 0.2% collagenase (Wako Chemicals), 25 units/mL deoxyribonuclease I (Sigma-Aldrich), 10 mM HEPES, and 1% penicillin/streptomycin]. Bone fragments were tapped with a mortar and pestle 50–100 times and filtered with a cell strainer (Falcon 2350) to remove debris and bone pieces. Cells were collected by centrifugation at 300×*g* at 4°C for 5 min, hemolyzed, and washed with FACS buffer (PBS supplemented with 2% FBS). Regarding LepR^+^MSC staining, BM cells were isolated by flushing from femurs and tibias, followed by enzyme digestion with collagenase and deoxyribonuclease I solution at 37°C for 20 min. After hemolysis, BM cells were washed with FACS buffer and filtered.

### FCM and cell sorting

FCM and cell sorting were performed using antibodies recognizing the following antigens: Cd45 (30-F11), Ter119 (TER-119), Pdgfrα (APA5), Sca1 (D7), Cd31(MEC13.3), Cd51 (HMαV-1), Cd24 (M1/69 or 30-F1), and the leptin receptor (goat polyclonal antibody). These antibodies were purchased from eBioScience, BioLegend, TONBO, and R&D Systems. Dead cells were eliminated by staining with 1 μg/mL propidium iodide (Sigma-Aldrich). PαS (Cd45^-^Ter119^-^Pdgfrα^+^Sca1^+^), LepR^+^MSC (Cd45^-^Ter119^-^Cd31^-^Sca1^-^LepR^+^), APC (Cd45^-^Ter119^-^Cd31^-^Cd24^-^Sca1^+^), OPC (Cd45^-^Ter119^-^Cd31^-^Pdgfrα^+^Sca1^-^), and Cd51^+^ stromal cells (Cd45^-^Ter119^-^Cd31^-^Cd51^+^) were defined as described elsewhere. The combination of antibodies was as follows:

**PαS+tFP635;** Biotin-anti-Pdgfrα, Fluorescein isothiocyanate (FITC)-anti-Sca1, Phycoerythrin (PE)-anti-Cd45, PE-anti-Ter119, and Allophycocyanin (APC)-Streptavidin. **PαS+GFP;** PE-anti-Pdgfrα, APC-anti-Cd45, APC-anti-Ter119, and Brilliant violet 421 (BV421)-anti-Sca1. **LepR**^**+**^**MSC+tFP635;** Biotin-anti-Pdgfrα, FITC-anti-Sca1, PE-anti-Cd45, PE-anti-Ter119, PE-anti-Cd31, and APC-Streptavidin. **LepR**^**+**^**MSC+GFP;** Biotin-anti-LepR, PE-anti-Cd31, APC-Cy7-anti-Cd45, APC-Cy7-anti-Ter119, PE-Cy7-anti-Sca1, and BV421-Streptavidin. **APC/OPC+GFP;** Biotin-anti-Cd24, PE-Streptavidin, APC-anti-Pdgfrα, APC-Cy7-anti-Cd45, APC-Cy7-anti-Ter119, PE-Cy7-anti-Cd31, and BV421-anti-Sca1. **Cd51**^**+**^**Stroma+GFP;** Biotin-anti-Cd51, PE-Streptavidin, APC-Cy7-anti-Cd45, APC-Cy7-anti-Ter119, and BV421-anti-Cd31. **Sorting of PαS subfractions;** Biotin-anti-Pdgfrα, FITC-anti-Sca1, PE-anti-Cd45, PE-anti-Ter119, and APC-Streptavidin.

Cell sorting and the analysis of tFP635 expression were performed on MoFlo (Beckman Coulter Life Sciences) equipped with a yellow-green laser, while other analyses were conducted on CytoFLEX (Beckman Coulter Life Sciences).

### MSC adherent culture

Sorted cells were cultured in growth medium (DMEM [Wako chemicals] supplemented with 20% FBS, 10 mM HEPES, and antibiotics) and incubated at 37°C with 5% CO_2_. Medium was replenished every 3–4 days.

### CFU-F assay

Approximately 500 sorted cells were seeded on a 100-mm dish in growth medium. A colony was defined as a cluster of 50 or more cells.

### Differentiation cultures

Adipogenic differentiation was induced using hMSC Adipogenic differentiation Medium Bullet Kit (LONZA) for 6 days, while osteogenic differentiation was induced with osteogenic media (DMEM supplemented 10 mM β-glycerophosphate, 50 μM L-ascorbic acid, and 1 mM dexamethasone) for 21 days. Cells were fixed with 4% paraformaldehyde for 10 min and stained with either Oil Red O or Alizarin Red according to standard protocol, and then images were acquired with a BZ-X700 fluorescent microscope (Keyence). After conversion to black and white image, the area with stains was quantified with ImageJ software.

### Quantitative RT-PCR

The total RNA was extracted using the RNeasy Micro Kit (Qiagen, Hilden, Germany) and reverse-transcribed by the PrimeScript Reverse Transcriptase (TaKaRa Bio, Japan) with a random hexamer primer. Real-time quantitative PCR was performed with the SYBR Green PCR Master Mix (ThermoFisher Scientific). The primer sequences used were as follows:

*Hprt1*, 5’-TCCTCCTCAGACCGCTTTT-3’, and 5’-CCTGGTTCATCATCGCTAATC-3’; *Fzd5*, 5’-CAGCAGGATCCTCCGAGA-3’, and 5’-CAGCACTCAGTTCCACACCA-3’; *Cxcl12*, 5’-CCAGAGCCAACGTCAAGCAT-3’, and 5’-CAGCCGTGCAACAATCTGAA-3’; *Kitl*, 5’-GGTAGCTAGTTCTATCCATGCGGT-3’, and 5’-CCTGTAAGGACTTTTCTGGAGAGTCT-3’; *FoxC1*, 5’-CACTCGGTGCGGGAAATGT-3’, and 5’-GGTACAGAGACTGACTGGCA-3’; *Lepr*, 5’-GTTCCAAACCCCAAGAATTG-3’, and 5’-TGATTCTGCATGCTTGGTAAA-3’; *Nes*, 5’-TCCCTTAGTCTGGAAGTGGCTA-3’, and 5’-GGTGTCTGCAAGCGAGAGTT-3’; *Cspg4*, 5’-GCAGAGGAGGTCTTGGTGAA-3’, and 5’-GGACATCTCGTGCTCATACAGA-3’.

### Statistical analysis

Statistical analyses were performed using Graph Pad Prism version 9. The significance of differences was measured by the Student’s *t* test. Data are shown as the mean ± SEM. Significance was taken at values of *p less than .05, ** *p* less than .01, *** *p* less than .005, **** *p* less than .0005, and ***** *p* less than .00005.

## Results

### Generation of BAC transgenic mice

**Fig. 1:**
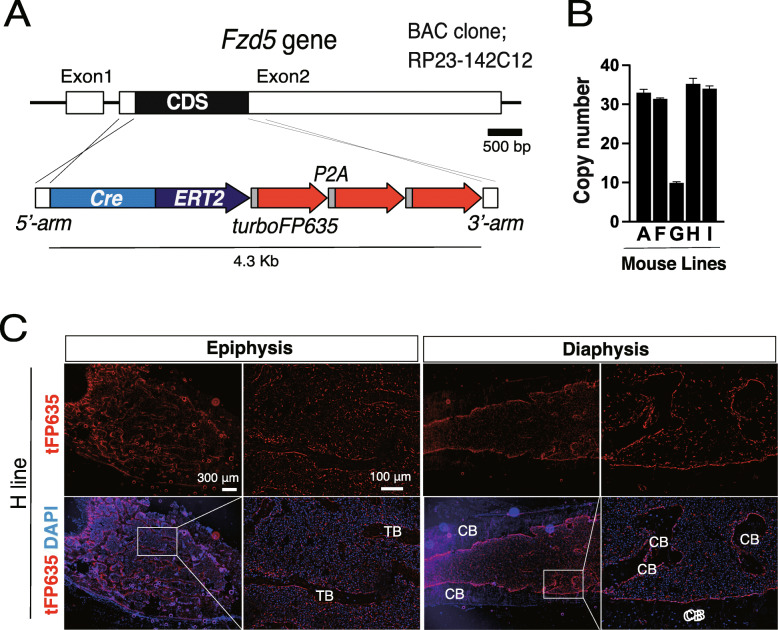
Generation of *Fzd5-CreERT-tFP635* mice using BAC transgenic technology. **A** The BAC modification strategy to mark *Fzd5*-expressing cells by tamoxifen-inducible Cre recombinase (CreERT) and the fluorescent protein, TurboFP635 (tFP635). *CreERT* and three tandem copies of *tFP635* cDNA were linked by P2A and inserted immediately after first ATG of the *Fzd5* gene in BAC using a homologous recombination technique. **B** The copy numbers of BAC DNA in the *Fzd5-CreERT-tFP635A*, *F*, *G*, *H*, and *I* lines were assessed by quantitative genomic PCR in triplicate. **C** tFP635 expression in the femur from *Fzd5-CreERT-tFP635H*. The femur was sectioned according to Kawamoto’s method. After 4′,6-diamidino-2-phenylindole (DAPI) staining, sections were observed under a fluorescent microscope. TB trabecular bone, CB cortical bone

We previously reported that FZD5 was preferentially expressed in MSC clones with a high proliferative capacity and multilineage differentiation capacity in humans. FZD5-mediated WNT signaling functions as a critical negative regulator of cellular senescence. The inhibition of FZD5 with short hairpin RNA led to premature cellular senescence [15]. These findings raised the question of whether the expression of Fzd5 also marks immature MSC in vivo. Therefore, we generated transgenic mouse strains using a BAC clone, in which the coding sequence of *Fzd5* was replaced with *CreERT* and 3 copies of *tFP635* cDNA linked with P2A sequences (Fig. 1A). Five *Fzd5-CreERT-tFP635* transgenic lines (F*zd5-CreERT-tFP635A*, *F*, *G*, *H*, and *I* line) from 10 independent founders were generated and backcrossed to C57BL6J mice more than 3 times to confirm transgene inheritance before assessments of BAC DNA copy numbers and an expression analysis of tFP635. Quantitative genomic PCR using primers, which amplify a 5′-UTR region in both the endogenous and exogenous *Fzd5* gene, revealed the copy numbers of BAC DNA (Fig. 1B).

### Localization of tFP635^+^ cells in the femur

To examine the anatomical localization of tFP635^+^ cells in BM, we performed a histological analysis of femur sections from *Fzd5-CreERT-tFP635H* and found that tFP635^+^ cells were abundant in the epiphysis and to a lesser extent in the diaphysis. tFP635^+^ cells showed two distinct localization patterns: cells lining the endosteum or those uniformly distributed to BM (Fig. 1C). We also observed the similar localization pattern of tFP635^+^ cells in the femur sections from *Fzd5-CreERT-tFP635I* (Additional file 1). These results demonstrated that at least two types of cells express tFP635 in *Fzd5-CreERT-tFP635*.

### Optimization of the MSC preparation protocol

Since the anatomical localization of BM-MSC in long bones, e.g., the tibia and femur, is unique to each MSC subtype, we optimized the protocol to prepare a single-cell suspension for a flow cytometric analysis (FCM) of two identified BM-MSCs, PαS (Cd45^-^Ter119^-^Pdgfrα^+^Sca1^+^), and LepR^+^MSC (Cd45^-^Ter119^-^Cd31^-^Sca1^-^Pdgfrβ^+^Lepr^+^). To achieve this, dissected long bones were initially divided into 2 parts, the bone ends (BE), and bone shaft, then the shaft was flushed out to obtain “marrow”. Residual bone shaft (bone), BE, and unfractionated whole bone (WB) were separately minced and digested with collagenase and subjected to FCM (Additional file 2A, B and C). The frequency of PαS was the highest in BE, and absolute number of PαS in BE was equivalent to that of WB, suggesting that PαS mainly resided in BE (Additional file 2B, D and E). Pdgfrα single-positive cells (PDSP) and Sca1 single-positive cells (SSP) were distributed in all four fractions to some extent, and the absolute number was the most abundant in WB (Additional file 2B, D and E). In contrast, LepR^+^MSC exclusively resided in marrow (Additional file 2C, D and E). Notably, we noticed the Lepr^+^ non-hematopoietic/endothelial (Cd45^-^Ter119^-^Cd31^-^Sca1^-^) cells contained both Pdgfrβ^+^ and Pdgfrβ^-^ cells. The latter was abundant in bone fraction or to some extent in BE and WB, whereas in the marrow, the Lepr^+^ cells were predominantly positive for Pdgfrβ (Additional file 2C). Based on these results, we prepared WB fractions from one leg to detect PαS and marrow from the other leg to identify LepR^+^MSCs that defined as Cd45^-^Ter119^-^Cd31^-^Sca1^-^Lepr^+^ cells in subsequent experiments.

### tFP635 expression in *Fzd5-CreERT-tFP635*

**Fig. 2:**
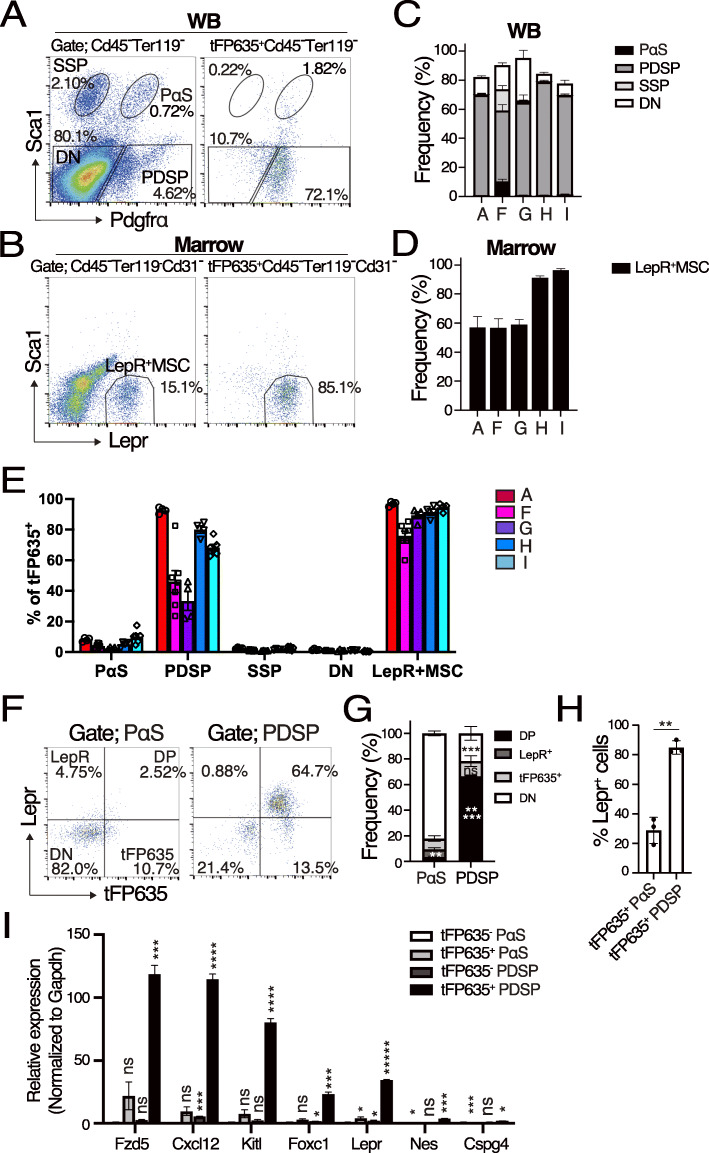
*Fzd5-CreERT-tFP635* marks LepR^+^MSC and some PαS in the long bones. The femurs and tibias were dissected from *Fzd5-CreERT-tFP635I.* A femur and tibia pair was used to prepare WB for PαS staining (**A**), while bone marrow (BM) was flushed out from the other pair for LepR^+^MSC staining (**B**). Frequencies of PαS, PDSP, SSP, and DN in tFP635^+^ non-hematopoietic cells (**C**) and that of LepR^+^MSCs in tFP635^+^ stromal cells (**D**) from the *Fzd5-CreERT-tFP635A* (*n*=4), *F* (*n*=7), *G* (*n*=4), *H* (*n*=4), and *I* line (*n*=6). **E** Frequencies of tFP635^+^ cells in PαS, PDSP, SSP, DN, and LepR^+^MSC from the *Fzd5-CreERT-tFP635A* (*n*=4), *F* (*n*=7), *G* (*n*=4), *H* (*n*=4), and *I* line (*n*=6). **F** Expression of Lepr and tFP635 in PαS and PDSP from *Fzd5-CreERT-tFP635I.* **G** Frequencies of Lepr^+^tFP635^+^ (DP), Lepr single-positive cells (Lepr^+^), tFP635 single-positive cells (tFP635^+^), and Lepr^-^tFP635^-^ (DN) in PαS (Left panel) and PDSP (right panel) (*n*=3). **H** Frequencies of Lepr^+^ cells in tFP635^+^PαS and tFP635^+^PDSP (*n*=3). **I** RT-qPCR analysis of the indicated genes in tFP635^-^PαS, tFP635^+^PαS, tFP635^-^PDSP, and tFP635^+^PαS. *Gapdh* was used to normalize the amount of input RNA. All data are shown as the mean ± SEM. *P < .05, **P < .01, ***P < .005, ****P < .0005, and ****P < .00005 by the Student’s *t* test

To test the expression pattern of tFP635, we conducted FCM of WB and marrow from *Fzd5-CreERT-tFP635I* mice. In the WB fraction, tFP635^+^ cells were mainly located in the PDSP region, followed by the double negative (DN) and PαS, and were negligible in the SSP region (Fig. 2A). In the marrow, approximately 90% of tFP635^+^ cells were in Sca1^-^ Lepr^+^ region (Fig. 2B). All five strains tested in the present study showed similar results with some variations (Fig. 2C, D). We noted that tFP635 was also expressed in some Cd45^+^ or Ter119^+^ hematopoietic cells (HC) (data not shown). In reverse gating, approximately 10% of PαS, 40-90% of PDSP, less than 1% of SSP/DN, and 90% of LepR^+^MSC (except for line F) were positive for tFP635 (Fig. 2E). In summary, cells expressing tFP635 largely overlapped with LepR^+^MSCs but less with PDSP. In other words, PDSPs contain more tFP635-negative cells than LepR^+^MSCs. So, we focused on tFP635-negative cells in the PDSPs. In an additional analysis (Fig. 2F, see also Additional file 3 for full FCM profiles), approximately 80% of PDSP were positive for tFP635 and most of them were also positive for Lepr, while the cells negative for tFP635 expression were predominantly negative for Lepr. These results showed that PDSP contains three subsets that are tFP635^+^LepR^+^MSC, tFP635^+^Lepr^-^, and tFP635^-^Lepr^-^. In PαS, more than 80% were negative for both tFP635 and Lepr (Fig. 2F–H). RT-qPCR revealed that tFP635^+^PDSP expressed not only Fzd5 and Lepr, but also the LepR^+^MSC markers, Cxcl12, Kitl, and FoxC1 [18, 19] (Fig. 2I). Collectively, these results showed that tFP635^+^PDSP are LepR^+^MSC. In addition, RT-qPCR of WB fractions revealed that Fzd5 expression is highest in PDSP, and followed by PαS, showing that the expression of tFP635 reflects endogenous Fzd5 expression (Additional file 4). Therefore, the expression of Fzd5 was mostly restricted to LepR^+^MSC and a part of PαS in the BM of *Fzd5-CreERT-tFP635*.

### Cell fate of Fzd5-expressing cells

**Fig. 3:**
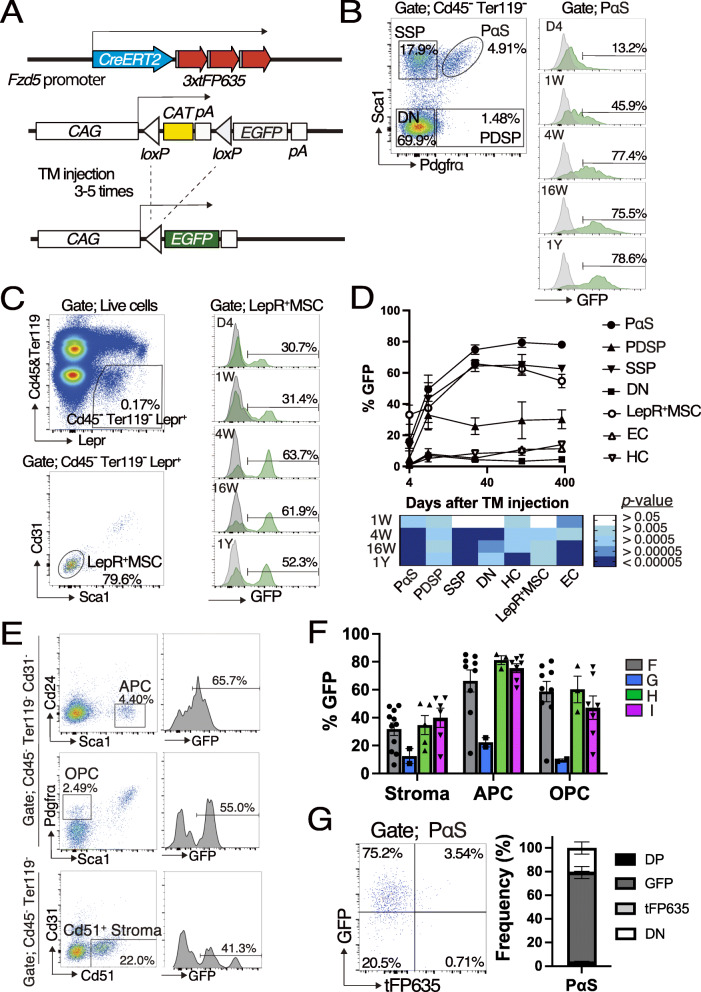
Tracing the lineage of *Fzd5*-expressing cells. **A** Strategy for the lineage tracing of *Fzd5*-expressing cells. **B**, **C** *Fzd5-GFP* were treated with TM at 8–12 weeks of age and the expression of GFP was analyzed in PαS (**B**) and LepR^+^MSC (**C**) at the indicated time points. **D** Time course changes in GFP positivity in PαS, PDSP, SSP, DN, LepR^+^MSC, EC, and HC are shown (*n*=5–10). The heat map shows *p* values against day 4. **E** Frequencies of GFP^+^ cells in APC, OPC, and Cd51^+^ stroma 16 weeks after the TM injection (*n*=9–11). **G** Expression of GFP and tFP635 in PαS from *Fzd5-GFP* 4 weeks after the TM injection (*n*=3). All data are shown as the mean ± SEM. *P < .05, **P < .01, ***P < .005, ****P < .0005, and ****P < .00005 by the Student’s *t* test

To trace the fate of *Fzd5*-expressing cells, mainly LepR^+^MSC and PαS, we crossed *Fzd5-CreERT-tFP635I* with *CAG-CAT-EGFP*, in which a *loxP*-flanked *CAT-pA* cassette impedes CAG promoter-driven GFP expression [16] and generated *Fzd5-CreERT-tFP635*; *CAG-CAT-EGFP* (hereafter referred to as *Fzd5-GFP*). In the *Fzd5-GFP*, the activation of CreERT irreversibly excised the cassette and induced the expression of GFP in *Fzd5*-expressing cells and their descendants (Fig. 3A). *Fzd5-GFP* were injected with TM for 3 or 5 consecutive days at between 8 and 12 weeks old to induce CreERT activity, and GFP expression was monitored in stromal fractions for 12 months (Fig. 3B–D). On day 4, approximately 10% of PαS were labeled with GFP, and GFP positivity and intensity progressively increased over time. The GFP positivity was maintained at approximately 80% for up to 12 months after the TM injection (Fig. 3B, D), whereas tFP635 along with CreERT was only expressed by a small portion of PαS cells (Fig. 2A, C, E). The proportion of GFP^+^ cells in SSP also increased with similar kinetics to PαS despite the lack of obvious tFP635 expression in SSP (Fig. 2A, C, E and Fig. 3D). In the marrow, approximately 30% of LepR^+^MSC expressed GFP by day 4, despite over 80% of LepR^+^MSC was positive for tFP635. GFP positivity slightly increased and was maintained at 60% for 12 months (Fig. 3C, D). It is known that TM does not necessarily work on all cells, this may explain the discrepancy with the results showing that LepR^+^MSC were largely positive for tFP635 (Fig. 2A–D). We also demonstrated that adipocyte progenitor cells (APC) [20], osteoblast progenitor cells (OPC) [20], and Cd51^+^ stromal cells were labeled with GFP at 16 weeks after the TM injection in all of the *Fzd5-CreERT-tFP635* lines tested (Fig. 3E, F). We note that *Fzd5-CreERT-tFP635F*, *G*, and *H* showed similar results with some variations according to copy number of BAC DNA (Additional file 5). We also analyzed the expression of tFP635 and GFP in the PαS from *Fzd5-GFP* mice 4 weeks after TM injection and showed that more than 70% of PαS cells were GFP^+^tFP635^-^ (Fig. 3G).

### *Lepr-tdTomato* also marks PαS subpopulation

**Fig. 4:**
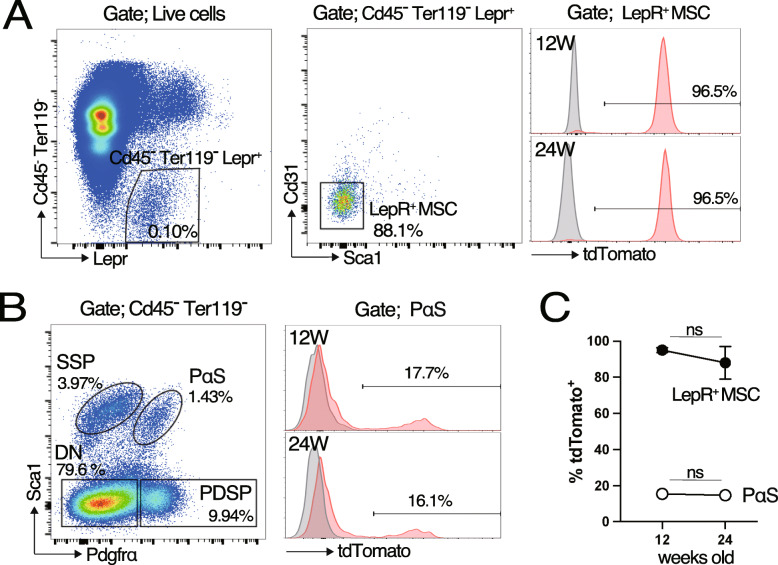
*LepR-tdTomato* labels LepR^+^MSC and some PαS in the long bones. Expression of tdTomato in LepR^+^MSC (**A**) and PαS (**B**) from *LepR-tdTomato* at 12 and 24 weeks of age. (**C**) Time course changes in tdTomato positivity in LepR^+^MSC and PαS are shown (*n*=3)

LepR^+^MSC have been identified as the primary source of bone and adipocytes in adult BM. *Lepr-Cre* marks LepR^+^MSC, and lineage tracing showed that LepR^+^MSC gave rise to osteoblasts and adipocytes in adult BM in vivo [7, 8]. Moreover, cell fate mapping with the *Ebf3-CreERT* strain revealed that CAR cells were self-renewing MSC in adult BM [21]. Similarly, we herein showed that *Fzd5-GFP* marked LepR^+^MSC with CreERT and tFP635. Therefore, we examined *Lepr-Cre*; *ROSA-CAG-LSL-tdTomato* (hereafter referred to as *Lepr-tdTomato*) mice at 12 and 24 weeks old to directly compare the identities of the cells labeled in these two different Cre strains (Fig. 4A–C). Consistent with previous findings, virtually all LepR^+^MSC were positive for tdTomato in 12-week-old *Lepr-tdTomato* mice, and the proportion of tdTomato^+^LepR^+^MSC was maintained for another 12 weeks (Fig. 4A, C). In contrast, tdTomato expression was observed in limited PαS cells, and the proportion of tdTomato-labeled cells was low even in 24-week-old mice (Fig. 4B, C), while the frequency of GFP^+^PαS progressively increased in *Fzd5-GFP* after the TM injection. The data, together with the result that tFP635^+^PαS cells were largely negative for Lepr, indicates that tFP635^+^PαS was distinct subpopulation from tdTomato^+^PαS.

### Fzd5^-^PDSP retains MSC properties

**Fig. 5:**
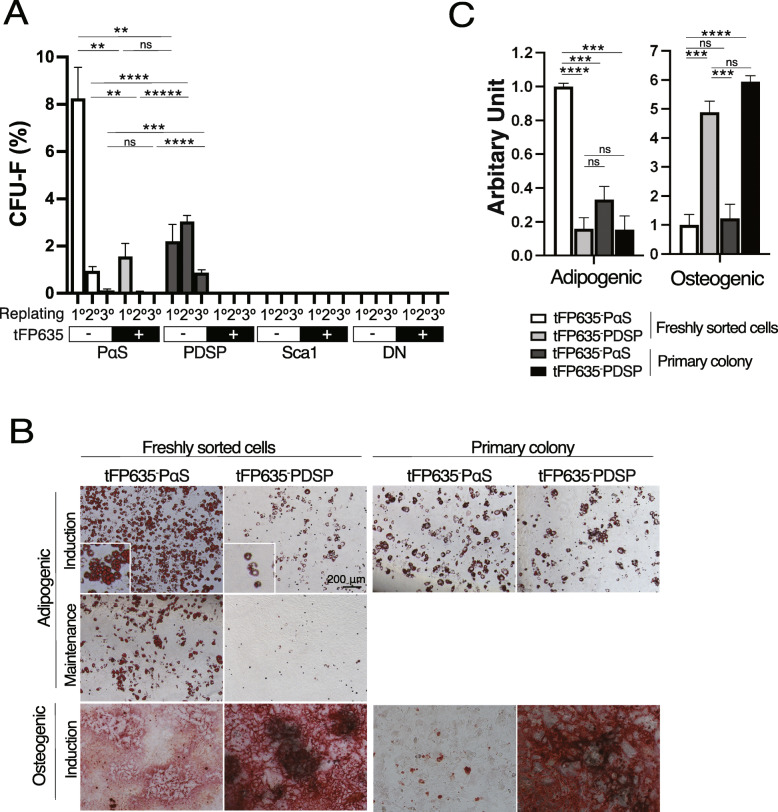
tFP635^-^PDSP retain secondary colony-forming activity. **A** Number of CFU-Fs 14 days after plating. Data are shown as the mean ± SEM (*n*=3). *P < .05; **P < .01; ***P < .005; ****P < .0005; *****P < .00005 by the Student’s *t* test. **B** Differentiation capacities of freshly sorted tFP635^-^PαS, tFP635^-^PDSP, and cells consisting of tFP635^-^PαS- or tFP635^-^PDSP-derived primary colonies (primary colony). **C** Quantification of Oil Red O (Adipogenic) and Alizarin Red (Osteogenic) staining

We investigated whether *Fzd5-CreERT-tFP635* enable the identification of a novel MSC fraction in BM. We fractionated non-hematopoietic cells from *Fzd5-CreERT-tFP635I* into eight subfractions according to the expression of Sca1, Pdgfrα, and tFP635 and tested their colony-forming unit-fibroblast (CFU-F) activities. Among the eight subfractions, tFP635^-^PαS exhibited the highest CFU-F activity, followed by tFP635^-^PDSP and tFP635^+^PαS in primary plating. tFP635^+^PDSP, which consists of LepR^+^MSC, had no apparent colony-forming capacity. Of note, tFP635^-^PDSP formed secondary colonies at a similar frequency to the primary colonies, even with marked reductions in the CFU-F activity of tFP635^-^PαS in secondary plating. Moreover, tFP635^-^PDSP formed colonies even in the third round of plating (Fig. 5A). These data suggest that tFP635^-^PDSP fraction contains more CFU-Fs with long-term replating capacity than tFP635^-^PαS fraction even though its frequency is low. We then examined the differentiation capacity of freshly sorted tFP635^-^PDSP and found that the cells accumulated lipid droplets less efficient than tFP635^-^PαS in adipogenic induction media (Fig. 5B, C). tFP635^-^PαS accumulated lipid droplets even in the maintenance media, which lacks adipogenic inducers but maintains adipocyte lineage cells, whereas tFP635^-^PDSP did not (Fig. 5B middle panels). In contrast, osteogenic differentiation was more efficient in tFP635^-^PDSP. Then, we dissociated the tFP635^-^PαS or tFP635^-^PDSP primary colonies with trypsin and assessed the differentiation capacity. The cells from tFP635^-^PDSP primary colonies differentiated into adipogenic cells as efficient as freshly sorted tFP635^-^PDSP, whereas tFP635^-^PαS greatly reduced the adipogenic capacity during culture in the colony assay condition (Fig. 5B, C). These data together indicated that cells with a colony-forming capacity in the tFP635^-^PDSP possess an adipogenic potential in addition to osteogenic potential.

## Discussion

We previously reported that the expression of FZD5 distinguished immature MSC clones with a high proliferative capacity and multilineage differentiation capacity from MSC clones with a limited replicative life span in vitro. This finding prompted us to investigate whether the expression of *Fzd5* also marks immature MSC in vivo, and, thus, we generated the mouse strain, *Fzd5-CreERT-tFP635*, which expresses CreERT and tFP635 in *Fzd5*-expressing cells. In *Fzd5-CreERT-tFP635*, the *Fzd5* promoter drove tFP635 to LepR^+^MSC and a small fraction of PαS. LepR^+^MSC showed adipogenic, osteogenic, and chondrogenic differentiation in vitro and in vivo and inducible lineage tracing using *Ebf3-CreERT* also revealed the lifelong self-renewal capacity of LepR^+^MSC in the adult stage [7, 8, 21]. Moreover, PαS retained tri-lineage differentiation and self-renewal capacities in vitro. Therefore, *Fzd5* promoter-driven tFP635 marks two types of previously identified MSC fraction in animal. Recent studies reported that the proliferation and lineage choice of MSC were, at least partially, regulated by systemic and local external signals [22, 23]. *Fzd5-CreERT-tFP635*, which marks a part of PαS and LepR^+^MSC, is a valuable tool for assessing the effects of environmental cues on MSC dynamics. This is the first reporter strain that traces the turnover of PαS in a TM-inducible manner.

*Lepr-tdTomato* also marks some PαS in addition to LepR^+^MSC. tFP635^+^PDSP and *Lepr-tdTomato*^+^LepR^+^MSC appeared to largely overlap because the majority of tFP635^+^PDSP expressed Lepr; however, we were unable to experimentally confirm this due to technical limitations. In contrast, only 25% of tFP635^+^PαS expressed Lepr. In *Fzd5-GFP,* tFP635^+^PαS accounts for approximately 10% of PαS, and the frequency of GFP^+^ cells progressively increased to approximately 80% in PαS and maintained for a year after the TM injection. In striking contrast, the proportion of tdTomato^+^ cells was kept low in PαS during our observation period. Theoretically, at least, a part of GFP^+^PαS cells were tFP635^+^PαS cells or progenies of tFP635^+^PαS cells at early time-point since GFP labeled cells are CreERT-expressing cells at the time of TM injection or their descendants. Therefore, it seems that *Fzd5-CreERT-tFP635* and *Lepr-tdTomato* marked distinct PαS subpopulations. However, we cannot exclude the possibility that some GFP^+^PαS cells were derived from tFP635^+^ cells outside of the PαS fraction in later time-point, e.g., tFP635^-^PDSP/ LepR^+^MSC or DN, since PαS was not the only cell expressing CreERT in the *Fzd5-CreERT-tFP635*. An approximately 20% of PαS cells were not labeled with GFP even after 1 year of chasing period in the TM-treated *Fzd5-GFP*, indicating that these PαS cells were quiescent and rarely replaced with GFP^+^PαS in steady-state or GFP^-^PαS cells were derived from non-PαS fraction which do not express the CreERT (discussed below). Development of the mouse strain, which express the Cre recombinase specifically in the PαS, will enable us to understand the hierarchical relationship between the PαS and other stromal fractions, e.g., LepR^+^MSC, tFP635^-^PDSP, or SSP.

We also exhibited that *Fzd5-CreERT-tFP635* facilitates screening for non-hematopoietic subfractions in the BM and identified tFP635^-^PDSP as a putative novel MSC fraction. The colony-forming activity of tFP635^-^PDSP was approximately 25% of tFP635^-^PαS in primary plating, but was sustained or slightly increased in secondary plating and formed a significant amount of tertiary colonies, whereas the CFU-F activities of tFP635^-^PαS and tFP635^+^PαS markedly decreased in secondary plating. Freshly sorted tFP635^-^PDSP rarely differentiated into an adipogenic lineage, whereas tFP635^-^PαS robustly accumulated lipid droplets. In contrast, tFP635^-^PDSP uniformly differentiated into an osteolineage more efficiently than tFP635^-^PαS. Of note, cells consisting of CFU-F colonies derived from tFP635^-^PDSP clearly retained adipogenic and osteogenic potentials, showing that only cells with a colony-forming capacity in the tFP635^-^PDSP fraction possessed an adipogenic potential in addition to osteogenic potential. Therefore, a small portion of tFP635^-^PDSP is MSC, while the remainders are unipotential cells capable of generating osteolineage cells, thus lineage-biased MSC or osteoblast progenitor. On the other hand, tFP635^-^PαS seems to be a heterogenous population consisting of a small portion of MSC and APC. This result is consistent with previous report showing that APC and OPC are immunophenotypically defined as Cd45^-^Ter19^-^Cd31^-^Pdgfrα^+^Cd24^-^ and Cd45^-^Ter19^-^Cd31^-^Pdgfrα^+^Sca1^-^, respectively [20]. FCM and expression analyses revealed that tFP635^-^PDSP expressed neither LepR^+^MSC nor *Nestin-GFP*^+^MSC markers, demonstrating that tFP635^-^PDSP were previously unidentified MSC fraction. However, clonogenic in vivo long-term transplantation analyses are needed to confirm this result.

In the E13.5 hindlimb, more than 90% of non-hematopoietic cells are Pdgfrα^+^Sca1^-^. Some Pdgfrα^+^ cells start to express Sca1 as development proceeds, and obvious PαS fraction emerged at E18.5 [24]. Moreover, lineage tracing with *Pdgfrα-CreERT* showed that cells exhibiting CFU-F activity in the CR fraction mostly originated from Pdgfrα^+^ cells [25]. Collectively, these results imply that Pdgfrα^+^Sca1^-^ is a precursor of PαS in fetal development. Similarly, it is worth to speculate that tFP635^-^PDSP is a bona fide MSC that give rise to PαS, and also other known MSC, in adults.

## Conclusions

Here, we generated the transgenic mouse strain (*Fzd5-CreERT-tFP635*) that express the *CreERT* and *tFP635* under the transcriptional control of *Fzd5* gene using BAC transgenic technique and showed that the mouse strain marks two different types of MSCs, PαS, and LepR^+^MSC.

## Supplementary Information

**Additional file 1. **Localization of tFP635^+^ cells in the femur from *Fzd5-CreERT-tFP635I* (related to Figure 1). The femur sections were stained with DAPI and observed under a fluorescent microscope. TB, trabecular bone; CB, cortical bone.

**Additional file 2. **Anatomical localization of PαS and LepR^+^MSC (related to Figure 2). (A) Schematic representation of the anatomical fractionation experiment. (B) After fractionation of the long bones and enzymatic digestion, a single-cell suspension was stained for PαS (B) or LepR^+^MSC (C). (D, E) Frequency (D) and absolute number (E) of PαS, PDSP, SSP, and LepR^+^MSC in each fraction. Data are shown as the mean ± SEM. *P < .05; **P < .01; ***P < .005; ****P < .0005; ****P < .00005 by the Student’s *t*-test.

**Additional file 3..** Gating strategy for analysis of Lepr and tFP635 expression in the PαS and PDSP (related to Figure 2). Expression of Lepr and tFP635 in the PαS or PDSP cells from indicated mouse was assessed by FCM.

**Additional file 4..** Fzd5 expression in the various BM fractions (related to Figure 2). RT-qPCR analysis of Fzd5 in PαS, PDSP, SSP and Cd45^+^ cells. Hprt1 was used to normalize the amount of input RNA. All data are shown as the mean ± SEM.

**Additional file 5. **Lineage analyses of *Fzd5-CreERT-tFP635F, G,* and *H* (related to Figure 3). Time course changes in GFP positivity in PαS, PDSP, SSP, DN, LepR^+^MSC, EC, and HC using *Fzd5-CreERT-tFP635F* (n=5-11), *G* (n=3-14), and *H* (n=4-5). Data are shown as the mean ± SEM.

## Data Availability

The data that support the findings of this study and materials are available from the corresponding author, SM, upon reasonable request

## Abbreviations

FZD5: Frizzled 5
BAC: Bacterial artificial chromosome
MSC: Mesenchymal stem/stromal cell
BM: Bone marrow
PαS: Pdgfrα^+^Sca-1^+^Cd45^−^Ter119^−^ subfraction
LepR^+^MSC: Leptin receptor-expressing MSCs
CRs: Collagenase-released cells
FCM: Flow cytometry
PDSP: Pdgfrα single-positive cell
SSP: Sca1 single-positive cell
APC: Adipocte prohenitor cell
OPC: Osteoblast progenitor cell
SCF: Stem cell factor
Kit: Kit ligand
Cxcl12: C–X–C Motif Chemokine Ligand 12
