# Supplementary figures and images for: Generation of a BAC transgenic mouse strain that expresses CreERT and a fluorescent protein under the transcriptional control of the Fzd5 locus
Source: Inflamm Regen. 2022 Mar 1;42:6. doi: 10.1186/s41232-022-00194-x (PMC8886790; doi:10.1186/s41232-022-00194-x)

Additional file 1

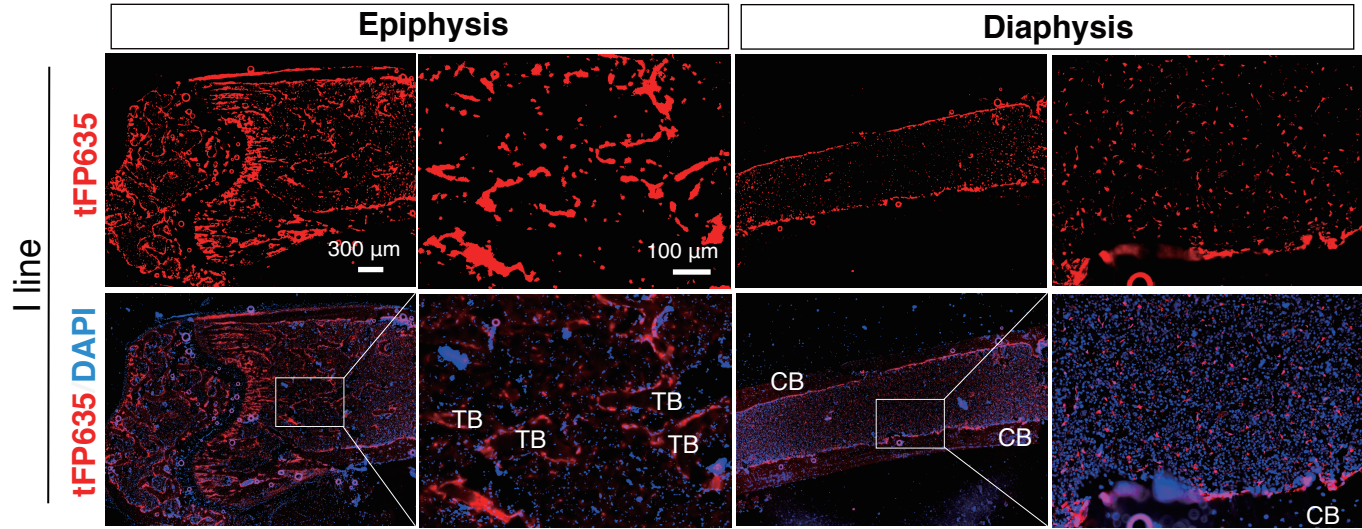

Supplement: Supplementary file 1 — Additional file 1. Localization of tFP635+ cells in the femur from Fzd5-CreERT-tFP635I (related to Figure 1). The femur sections were stained with DAPI and observed under a fluorescent microscope. TB, trabecular bone; CB, cortical bone. [file 41232_2022_194_MOESM1_ESM.pdf]

Additional file 2

A

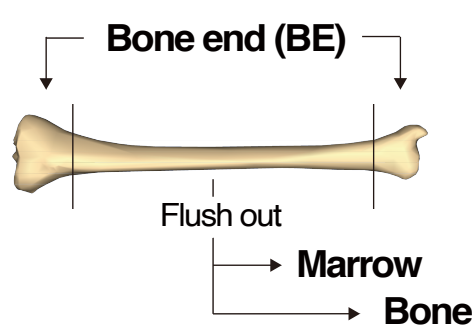

B

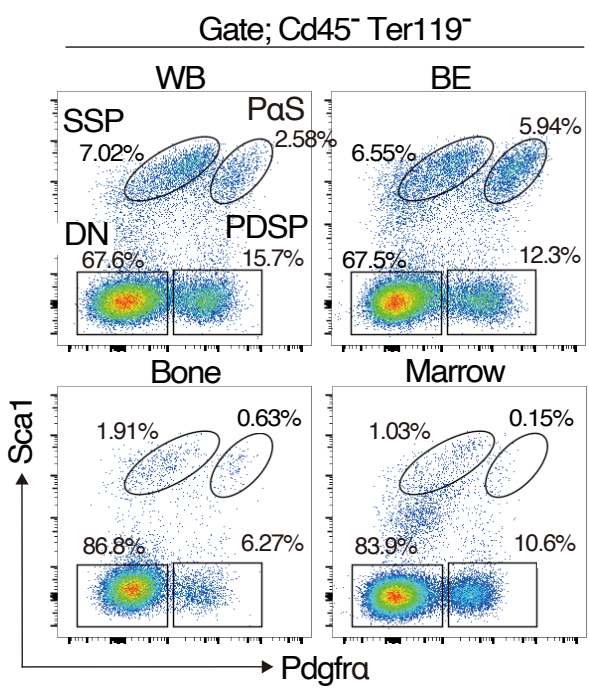

C

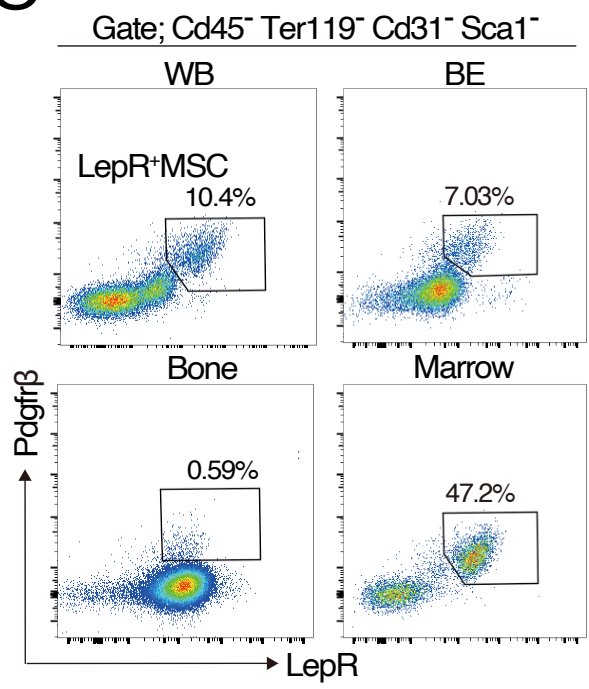

D

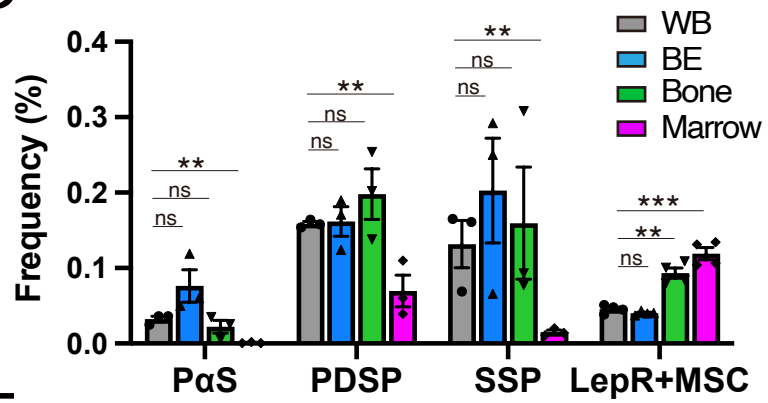

E

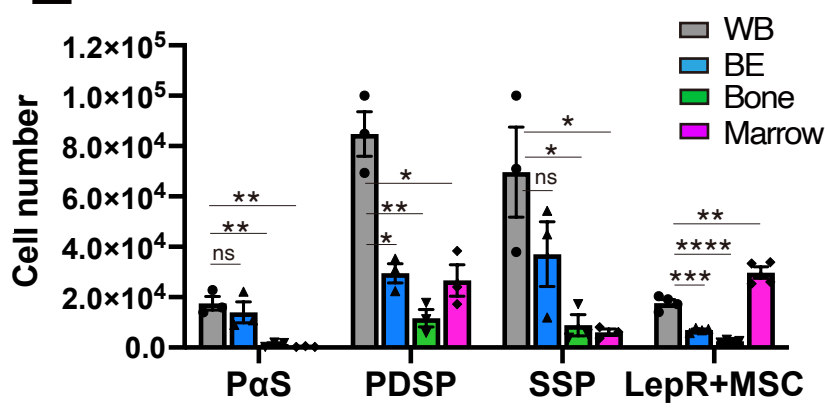

Supplement: Supplementary file 2 — Additional file 2. Anatomical localization of PαS and LepR+MSC (related to Figure 2). (A) Schematic representation of the anatomical fractionation experiment. (B) After fractionation of the long bones and enzymatic digestion, a single-cell suspension was stained for PαS (B) or LepR+MSC (C). (D, E) Frequency (D) and absolute number (E) of PαS, PDSP, SSP, and LepR+MSC in each fraction. Data are shown as the mean ± SEM. *P < .05; **P < .01; ***P < .005; ****P < .0005; ****P < .00005 by the Student’s t-test. [file 41232_2022_194_MOESM2_ESM.pdf]

Additional file 3

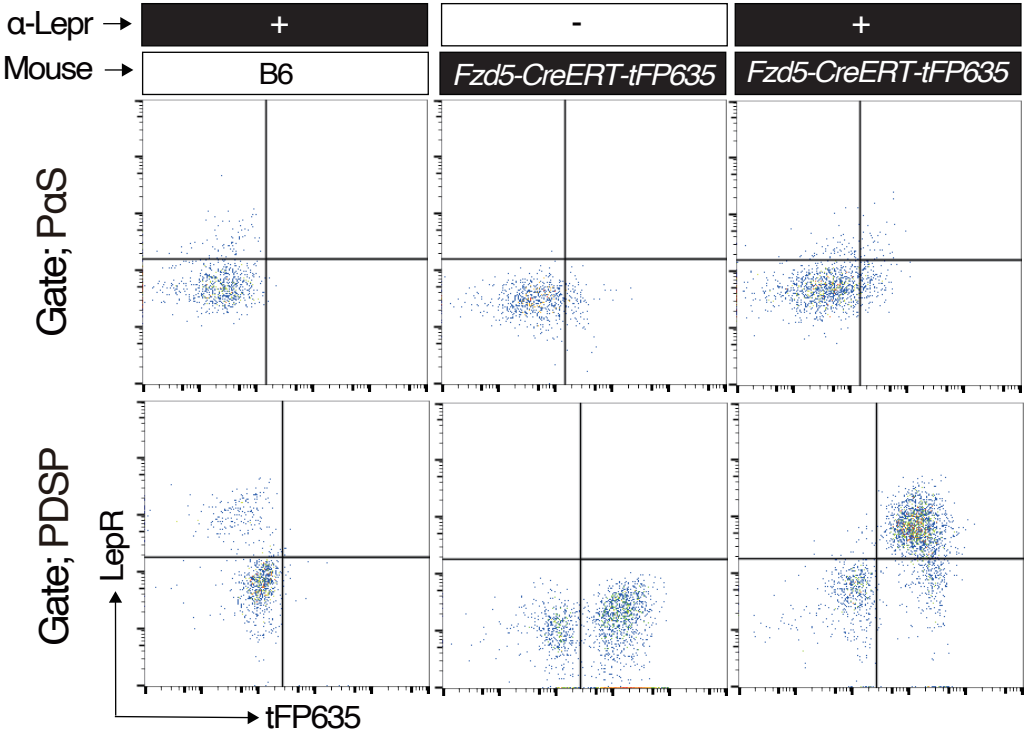

Supplement: Supplementary file 3 — Additional file 3.. Gating strategy for analysis of Lepr and tFP635 expression in the PαS and PDSP (related to Figure 2). Expression of Lepr and tFP635 in the PαS or PDSP cells from indicated mouse was assessed by FCM. [file 41232_2022_194_MOESM3_ESM.pdf]

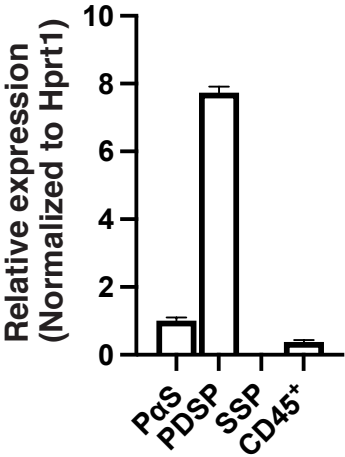

Supplement: Supplementary file 4 — Additional file 4.. Fzd5 expression in the various BM fractions (related to Figure 2). RT-qPCR analysis of Fzd5 in PαS, PDSP, SSP and Cd45+ cells. Hprt1 was used to normalize the amount of input RNA. All data are shown as the mean ± SEM. [file 41232_2022_194_MOESM4_ESM.pdf]

Additional file 5

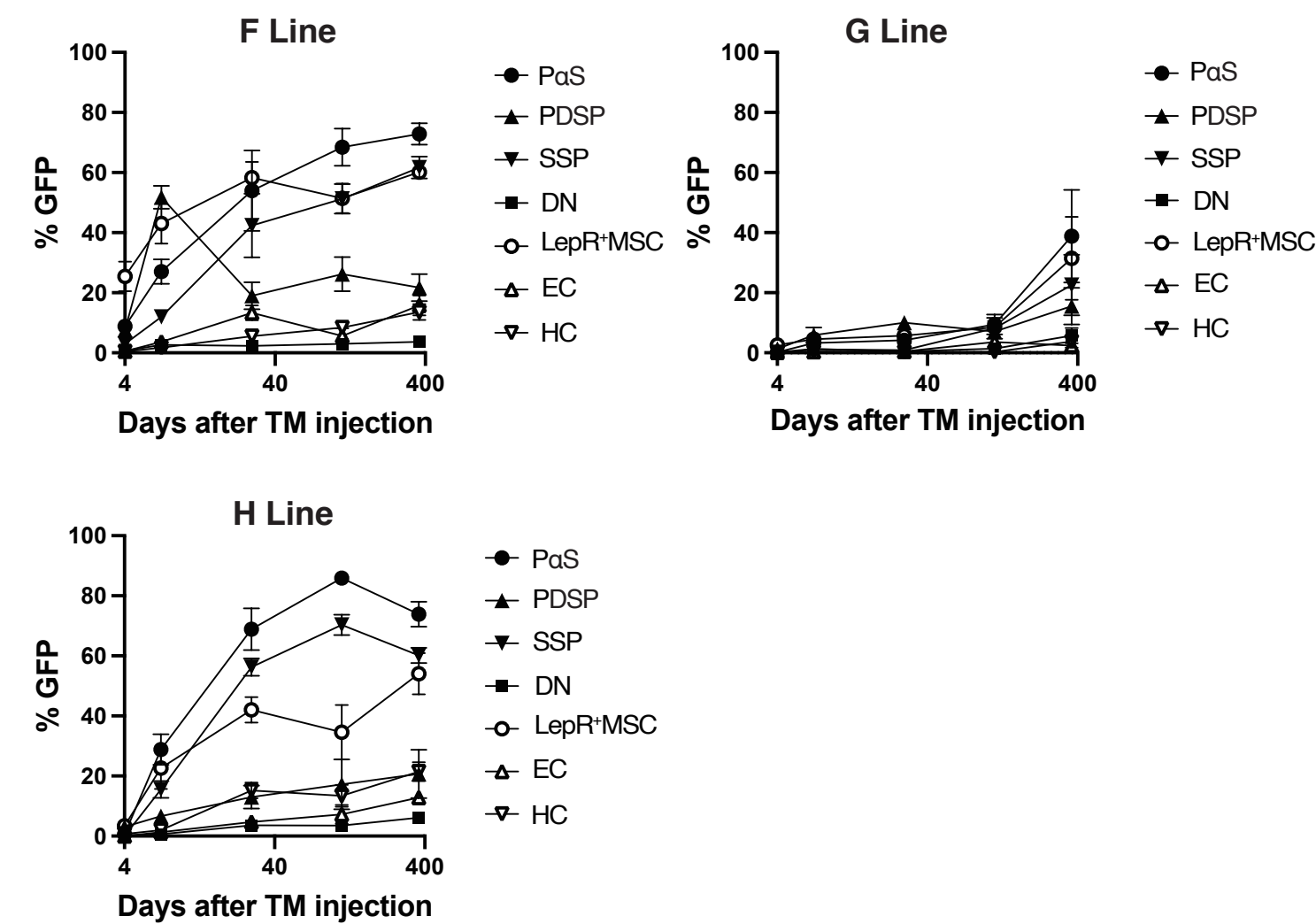

Supplement: Supplementary file 5 — Additional file 5. Lineage analyses of Fzd5-CreERT-tFP635F, G, and H (related to Figure 3). Time course changes in GFP positivity in PαS, PDSP, SSP, DN, LepR+MSC, EC, and HC using Fzd5-CreERT-tFP635F (n=5-11), G (n=3-14), and H (n=4-5). Data are shown as the mean ± SEM. [file 41232_2022_194_MOESM5_ESM.pdf]
